# Families Created by Egg Donation: Parent–Child Relationship Quality in Infancy

**DOI:** 10.1111/cdev.13124

**Published:** 2018-07-17

**Authors:** Susan Imrie, Vasanti Jadva, Simon Fishel, Susan Golombok

**Affiliations:** ^1^ University of Cambridge; ^2^ CARE Fertility

## Abstract

Increasing numbers of children are being born through egg donation and thus do not share a genetic relationship with their mother. Parent–infant relationship quality was examined in 85 egg donation families and a comparison group of 65 in vitro fertilization families (infant *M *=* *11 months). Standardized interview and observational measures were used to assess mother–infant and father–infant relationship quality at the representational and behavioral levels. Few differences were found between family types in parents’ representations of the parent–infant relationship. Differences were found between family types in the observational assessment of mother–infant relationship quality, indicating less optimal interactions in egg donation families. Findings suggest that egg donation families function well in infancy overall, but there may be subtle yet meaningful differences in mother–infant interaction quality.

Egg donation is an increasingly common fertility treatment used by women who are unable to conceive using their own eggs (Lutjen et al., 1984). In cases of heterosexual couples using egg donation, an embryo is created using a donor egg and the father's sperm. The resulting child is thus genetically related to the father but not to the mother who raises the child. Egg donation is particularly widely used in the United States, with over 3,000 live births resulting from fresh donor egg cycles started in 2015, of which 27% were twin births (Centers for Disease Control and Prevention, 2017). In the United Kingdom, over 1,400 babies were born following treatment with in vitro fertilization (IVF) and donor eggs in 2016, with just over half of these cycles undertaken by women aged 40 or over (Human Fertilisation and Embryology Authority, 2018).

Despite its growing popularity as a family‐building option, little is known about the quality of parent–child relationships in families formed in this way. Empirical evidence on family functioning in egg donation families has come almost exclusively from two longitudinal studies. The European Study of Assisted Reproduction Families compared egg donation, sperm donation, IVF, and adoptive families when children were aged 3–8 (Golombok, Murray, Brinsden, & Abdalla, 1999) and 12 years (Murray, MacCallum, & Golombok, 2006). The UK Longitudinal Study of Assisted Reproduction Families compared family functioning in egg donation, sperm donation, and natural conception families when children were aged 1, 2, 3, 7, 10, and 14 years (Golombok, Blake, Casey, Roman, & Jadva, 2013; Golombok, Ilioi, Blake, Roman, & Jadva, 2017; Golombok, Jadva, Lycett, Murray, & MacCallum, 2005; Golombok et al., 2004, 2006, 2011). Both studies found egg donation families to be functioning well in terms of quality of parenting, parents’ psychological health, and child adjustment, although both relied on parents’ interview data as a means of assessing parent–child relationship quality during early phases of the studies and included no observational assessments of parent–child interactions before age 7. Thus, evidence using in‐depth measures of parent–child relationships in the earlier years remains limited.

Infancy has been a focus of developmental psychology for over half a century, with the quality of the parent–infant attachment relationship identified as a crucial factor in influencing child socioemotional development across a range of outcomes (Bowlby, 1982), including externalizing psychopathology (Fearon, Bakermans‐Kranenburg, van IJzendoorn, Lapsley, & Roisman, 2010) and social competence (Groh, Fearon, van IJzendoorn, Bakermans‐Kranenburg, & Roisman, 2017). Factors identified as important antecedents of individual differences in attachment security include parental sensitivity, parental reflective functioning, and parental representations of the parent–child relationship.

Parental sensitivity, defined as a parent's ability to accurately perceive and interpret their child's cues and respond appropriately to these signals (Ainsworth, Blehar, Waters, & Wall, 1978), has been confirmed in meta‐analyses as playing a causal role in the development of both mother–infant and father–infant attachment security (Verhage et al., 2016). A growing body of work has suggested that parental reflective functioning (Slade, 2005)—a parent's ability to reflect on their own and their child's mental states and link them to their own and their child's behaviors—may underlie a parent's ability to provide sensitive care to their infant (Stacks et al., 2014). In low‐risk samples of mothers, reflective functioning has been found to be associated with infant–mother attachment security (Slade, Grienenberger, Bernbach, Levy, & Locker, 2005) and parenting behaviors (Rosenblum, McDonough, Sameroff, & Muzik, 2008). To date, no studies have examined parental reflective functioning in fathers with infants.

Parental representations of the parent–child relationship—a set of specific expectations about how their child will interact with them—are also thought to be fundamental to the development of the caregiving relationship (George & Solomon, 1996). These representational models determine access to particular feelings and thoughts about the child and guide parents’ behaviors and expectations within the relationship (Slade, Belsky, Aber, & Phelps, 1999). Mothers’ representations of the mother–infant relationship have been found to be related to infant–mother attachment security in nonclinical samples (Zeanah, Benoit, Hirshberg, Barton, & Regan, 1994) and to parent and infant interactive behaviors (Korja et al., 2010; Rosenblum, McDonough, Muzik, Miller, & Sameroff, 2002). Similarly, fathers’ attachment representations of the infant at 6 months predicted the quality of father–infant interaction at 24 months (Hall et al., 2014). The only study to examine egg donation parents’ representations of the parent–child relationship did so when children were aged 2 (Golombok et al., 2005). Egg donation mothers were found to represent the relationship as higher in joy, compared with sperm donation and natural conception mothers, and lower in overprotectiveness than sperm donation mothers. No group differences were found in fathers’ representations of the father–child relationship.

Concerns about the parent–infant relationship in egg donation families have centered primarily on the absence of a genetic connection between the mother and child. These concerns have arisen largely from dominant Euro‐American societal narratives which prioritize genetic ties over social relatedness in the creation and maintenance of kin relationships (Freeman, 2014), with survey data consistently finding that Euro‐American heterosexual couples demonstrate a preference for genetically related families over families built through other means (Daniluk & Koert, 2012).

Although adoption and egg donation differ in several fundamental ways, first that egg donation infants are born into the families who raise them, and second, that egg donation mothers give birth to their child, research on the transition to adoptive parenthood offers a useful reference point in so far as it highlights the early challenges and complexities involved in preparing for, and parenting, a genetically unrelated child. Within both the clinical literature on infertility and the adoption literature, the decision to pursue nongenetic parenthood is commonly framed as involving a loss of an imagined genetically related child (Cudmore, 2005; Daniluk & Hurtig‐Mitchell, 2003) and a reconstruction of conceptualizations of parenthood (Goldberg, Downing, & Richardson, 2009).

The decision to adopt may provide couples with a renewed sense of hope, purpose, and fulfillment (Daniluk & Hurtig‐Mitchell, 2003), and in terms of later attachment security, few differences have been found between early‐adopted infants and their nonadopted peers (van den Dries, Juffer, van IJzendoorn, & Bakermans‐Kranenburg, 2009). However, the period of transition to adoptive parenthood may present parents with specific challenges, including uncertainties around parental role definition (Kirk, 1964), feeling additional pressure to be “perfect parents” having waited so long for parenthood, and a lack of social support (McKay & Ross, 2010). In a study of 90 adoptive parents 2 years after placement, one‐third of parents reported a slow or “challenging” initial bonding process with their child, attributed to factors including the suddenness of the transition to parenthood and not feeling entitled to parent the child (Goldberg, Moyer, & Kinkler, 2013). Adoption‐related stigma is also a common experience for many new adoptive parents (Goldberg, Kinkler, & Hines, 2011). Although in egg donation families it is only the mother who lacks a genetic link to her child, fathers through egg donation may also be affected by stigma associated with nongenetic parenthood. Studies of assisted reproduction parents consistently find that many genetically related parents choose not to disclose donor conception to the child for fear of difficulties in the relationship between the child and the genetically unrelated parent, suggesting that genetic parents are not immune from concerns about stigmatization (Golombok et al., 2004).

In addition to having to consider the significance of parenting a genetically unrelated child, new egg donation parents are also likely to have experienced an extended period of infertility and fertility treatment prior to parenthood. Studies examining the effects of fertility treatment on mother–infant relationship quality have found few differences between families created through IVF and natural conception families (Gibson, Ungerer, McMahon, Leslie, & Saunders, 2000). However, there are suggestions of a cumulative effect of repeated treatment cycles on mothers’ sense of self‐efficacy with their infants at 4 months (McMahon, Ungerer, Tennant, & Saunders, 1997). Studies examining the emotional consequences of fertility treatment have suggested that when pregnancy is achieved after a long period of treatment, couples may not have had any opportunity to restore their psychological reserves before beginning the transition to parenthood and that negative feelings related to infertility could persist into the first year (Redshaw, Hockley, & Davidson, 2007). That repeated IVF cycles may have a cumulative negative effect on adjustment to parenthood is of particular relevance to egg donation families, who are likely to have experienced more treatment cycles than IVF parents, and have been identified as experiencing high levels of distress while awaiting treatment (Carter et al., 2011).

Since the removal of donor anonymity in the United Kingdom in 2005, patients pursuing egg donation treatment choose between identity‐release donation (where the donor is unknown to the recipient, but any resultant child may access identifying donor information at age 18) or known donation (where the donor is known to the recipient, e.g., a sister). A trend away from anonymous donation, and toward recommending identity‐release donation, has also been found among American fertility professionals (Speier, 2017). Identity‐release donation may pose distinct challenges to the parent–infant relationship, and its effects on family functioning in the current generation of egg donation families are unknown as all previous studies of egg donation families have comprised those who used either anonymous or known donation.

Egg donation parents reported choosing anonymous donation (i.e., where the donor's identity will never be known) over known donation as it enabled them to establish clear psychological boundaries between the donor and recipient family, minimize the link between the donor and child, and protect the mother–child relationship (Laruelle, Place, Demeestere, Englert, & Delbaere, 2011). Known donation, in contrast, was viewed as complicating the donor–child relationship and undermining the recipient's ability to feel secure in her role of mother (Greenfeld & Klock, 2004). Identity‐release donation occupies an uncertain middle ground between anonymous and known donation, in that recipients know little about the donor at the time of treatment, but they, and their child, will have access to identifying information, and potentially contact, with the donor in the future. Given that anonymous donation is often chosen by parents due to the wish to establish explicit boundaries between the donor and recipient family, and to help the mother feel secure in her parenting role, it could be argued that identity‐release donation may be viewed as a less secure and reassuring option, as it removes the explicit boundaries provided by anonymous donation, both psychologically and in practical terms. It is possible that identity‐release donation may negatively influence family functioning as the donor may be perceived as an ongoing presence within the family, thus posing a threat to the mother–child relationship (Lampic, Svanberg, & Sydsjö, 2014), or by the prospect of identity‐release adding an extra layer of complexity to parents’ disclosure decisions (Freeman, Zadeh, Smith, & Golombok, 2016). These concerns may affect parents’ thoughts and feelings about their parental role, in particular their sense of entitlement and confidence in their position as the child's parents and the future security of their family unit but may be particularly salient for mothers.

Little research on egg donation families has examined family functioning using observational or representational measures of the quality of the parent–child relationship, and none has done so in infancy. This study offers the first assessment of the quality of parent–infant relationships in families formed through egg donation and the first study worldwide of egg donation families who have used identity‐release donation. Although egg donation families have generally been found to be functioning well in early childhood when assessed using interview measures of family functioning, representational and observational measures may offer more detailed information about the quality of the parent–infant relationship. It is likely that the transition to parenthood is a time at which egg donation parents may be most affected by the mother's adjustment to nongenetic parenthood, their earlier experiences of fertility treatment, and any concerns about the lack of donor anonymity. It was, thus, hypothesized that egg donation mothers and fathers would show poorer parent–infant relationship quality when assessed at both the representational and behavioral levels, in comparison with IVF families in which both parents were genetically related to the child.

## Method

### Participants

Eighty‐five families (85 mothers, 67 fathers) created through egg donation and IVF and a comparison group of 65 families (65 mothers, 38 fathers) created through IVF using the parents’ own gametes participated in the study. Infants were aged 6–18 months. Of the egg donation families, 73 had used identity‐release donation and 12 had used known donation. Families were recruited through 12 fertility clinics in the United Kingdom. In order to maintain confidentiality, all families were initially contacted by the clinics and invited to send their contact details to the researchers in order to receive further information about the study. Clinics were asked to write to two‐parent heterosexual families with a child born in the previous 3–12 months, conceived through privately funded IVF treatment. Four hundred nineteen families were contacted and 190 returned contact details to the researchers, giving a response rate of 45%. Of the 110 egg donation families who returned contact details, 99 were eligible for the study, of whom 85 participated, giving a participation rate of 86%. Eighty IVF families returned contact details, of whom 72 were eligible. Of those, 63 families participated in the study, giving a participation rate of 88%. Two additional IVF families were recruited through snowballing.

Families with twins were included in the study, first, in order not to unnecessarily restrict the sample size of this hard‐to‐reach population, and second because multiple pregnancies are a common outcome of fertility treatment (Human Fertilisation and Embryology Authority, 2018).

As shown in Table 1, there was a significant difference between family types in mothers’ age, *t*(148)* *=* *7.87, *p *<* *.001, reflecting the older age of egg donation mothers (*M *=* *42.45 years) than IVF mothers (*M *=* *36.95 years). Egg donation fathers (*M *=* *43.26 years) were also significantly older than IVF fathers (*M *=* *40.35 years), *t*(148)* *=* *2.87, *p *=* *.01.

**Table 1:** Family Sociodemographic and Fertility Treatment Characteristics by Family Type

	Egg donation (*N* = 85)		IVF (*N *= 65)		Independent samples *t* test		
	*M*	*SD*	*M*	*SD*	*t*	*p*	*d*
Mother's age (years)	42.45	4.27	36.95	4.20	7.87	< .001	1.30
Father's age (years)	43.26	6.23	40.35	6.02	2.87	.01	0.47
Child's age (months)	11.34	2.15	9.68	1.97	4.88	< .001	0.80
Relationship (years)	12.06	5.25	11.89	4.70	0.21	.83	0.03

	*N* (%)	*N* (%)	Chi‐square		
			χ^2^	*df*	*p*
Sex of child					
Female	40 (47)	27 (42)	0.45	1	.50
Male	45 (53)	38 (58)			
Multiple rate					
Singleton	73 (86)	56 (86)	0.01	1	.94
Twin pair	12 (14)	9 (14)			
No. of siblings					
0	60 (71)	21 (32)	21.73	1	< .001
1 or more	25 (29)	44 (68)			
Mother's education					
School education	20 (27)	20 (32)	0.43	1	.51
Higher education	55 (73)	43 (68)			
Father's education					
School education	27 (36)	21 (33)	0.15	1	.70
Higher education	47 (64)	42 (67)			

	*M*	*SD*	*M*	*SD*	Mann–Whitney *U* test		
					*U*	*p*	*d*
Fertility treatment							
Decision to have child (years)	6.41	2.97	4.15	2.64	1,433.00	< .001	0.90
No. IVF cycles to conceive child	3.32	2.22	1.78	1.15	1,558.00	< .001	0.80

Note

IVF = in vitro fertilization.

There was a significant difference between groups in the child's age, *t*(148)* *=* *4.88, *p *<* *.001, with egg donation infants (*M *=* *11.34 months) older than IVF infants (*M *=* *9.68 months). There were similar proportions of male and female infants in each family type, χ^2^(1)* *=* *0.45, *p *=* *.50, and similar proportions of twins in each group, χ^2^(1)* *=* *0.01, *p *=* *.94. A significantly higher proportion of egg donation children had no siblings, χ^2^(1)* *=* *21.73, *p *<* *.001.

The majority of mothers (133, 96%) and fathers (127, 93%) identified their ethnic group as “white British/white Irish.” There was no significant difference between family types in the length of parents’ relationships, *t*(148)* *=* *0.21, *p *=* *.83, mothers’ educational level, χ^2^(1)* *=* *0.43, *p *=* *.51, or fathers’ educational level, χ^2^(1)* *=* *0.15, *p *=* *.71.

Families also differed significantly in their fertility treatment history. Egg donation parents had been trying to conceive the target child for longer than IVF parents (*U *=* *1,433.00, *p *<* *.001) and had undergone more IVF cycles (*U *=* *1,558.00*, p *<* *.001).

### Procedure

One of two psychologists visited the families at home, and written informed consent was obtained from each parent. Each parent was administered a video‐recorded observational assessment of parent–child interaction and an audio‐recorded standardized interview that lasted approximately 1.5 hr. Ethical approval was granted by the University of Cambridge Psychology Research Ethics Committee. Data were collected between October 2013 and June 2015.

### Measures

#### The Quality of the Parent–Infant Relationship

The quality of the parent–infant relationship was assessed using (a) a representational measure of the parent–infant relationship and (b) an observational measure of parent–infant interaction.

#### Representational Measure

Mothers and fathers were interviewed separately using an adaptation of the Parent Development Interview (PDI; Aber, Slade, Berger, Bresgi, & Kaplan, 1985), adapted by Henderson, Steele, and Hillman (2007). The PDI is a semistructured interview that assesses parents’ representations of the parent–child relationship. The PDI is derived from attachment theory and is based on the view that a parent's feelings and thoughts about their child influence their parenting behaviors. Parents are asked to describe themselves as a parent, their child, and their own and their child's experiences in moments of relatedness and interaction.

PDIs were transcribed verbatim and coded using a system developed by Henderson et al. (2007) which yields codes for the parent's representation of themself as a parent (parent affective experience codes), the parent's representation of the child (child affective experience codes), and reflective functioning.

The parent affective experience codes were (a) *degree of anger*, measuring the degree to which the parent is angry in the relationship (rated from 1 “none/minimal anger felt” to 4 “high anger felt”; (b) *expression of anger*, measuring the extent to which expressions of anger are present in the relationship (rated from 1 “no/minimal anger shown” to 4 “high anger shown”); (c) *need for support*, assessing the parent's acknowledgment of need for support (rated from 1 “none/minimal feelings of needing support” to 4 “high feelings of needing support”); (d) *satisfaction with available support*, assessing satisfaction with the level of support available to them (rated from 1 “no/minimal satisfaction” to 4 “high satisfaction”); (e) *guilt*, measuring the extent to which parental guilt is present in the relationship (rated from 1 “none/minimal guilt” to 4 “high guilt”); (f) *joy/pleasure*, assessing the parent's ability to express feelings of joy in relationship to and with the child (rated from 1 “none/minimal acknowledgment of joy” to 4 “high acknowledgment of joy”); (g) *competence*, measuring how well the parent is coping with the child (rated from 1 “low competence” to 4 “high competence”); (h) *parental confidence*, assessing the parent's sense of their own competence (rated from 1 “none/minimal confidence” to 4 “high confidence”); (i) *level of child focus*, measuring the degree to which the parent is focused on the needs of the child as compared with their own emotional needs (rated from 1 “none/minimal level of child focus” to 4 “high level of child focus”); (j) *disappointment/despair*, assessing the extent to which the parent expresses disappointment with being a parent (rated from 1 “none/minimal disappointment” to 4 “high despair”); (k) *warmth*, measuring the amount of warmth the parent feels toward the child (rated from 1 “none/minimal warmth” to 4 “high warmth”); (l) *attachment awareness and promotion*, assessing the parent's understanding of the attachment issues for their child and their ability to behave in ways that will promote the child's attachment to them (rated from 1 “none/minimal attachment awareness” to 4 “high attachment awareness”); (m) *hostility*, measuring hostile feelings toward the child (rated from 1 “none/minimal hostility” to 4 “high hostility”).

The child affective experience codes, used to assess the parent's representation of the child, were (a) *child anger*, assessing the degree to which the parent represents the child as experiencing or expressing anger (rated from 1 “none/minimal child anger” to 4 “high child anger”); (b) *child happiness*, measuring the degree to which the parent represents the child as happy and contented as distinct from the parent–child relationship (rated from 1 “none/minimal child happiness” to 4 “high child happiness”); (c) *child controlling/manipulating*, assessing the degree to which the parent represents the child as attempting to control the parent and their interactions (rated from 1 “none/minimal child controlling/manipulating” to 4 “high child controlling/manipulating”); (d) *child affection*, measuring the extent to which the child shows and accepts physical affection in relation to the parent (rated from 1 “none/minimal child affection” to 4 “high child affection”); (e) *child rejection*, assessing the extent to which the parent feels rejected by the child either emotionally or practically (rated from 1 “none/minimal child rejection” to 4 “high child rejection”).

Global codes included (a) *parental reflective functioning*, measuring the extent to which the parent can reflect on the child and the relationship (rated from 1 “none/minimal reflection” to 4 “high reflection”); (b) *coherence*, assessing the overall coherency of ideation and feeling in the parent's representation of the child (rated from 1 “none/minimal coherence” to 4 “high coherence”); (c) *richness of perceptions*, measuring the poverty or richness of the caregiver's perceptions of the child and the relationship with the child (rated from 1 “no/minimal richness of perception” to 4 “high richness of perception”).

To establish interrater reliability, 49 (33%) randomly selected mothers’ transcripts were coded by a second rater. Intraclass correlation coefficients ranged from .79 to .99.

#### Observational Measure

A free play task was used to obtain an observational assessment of mother–infant and father–infant interaction. Each parent was instructed to play with the infant for 10 min in whatever manner and using whichever toys, they used when they usually played together. The session was video recorded and coded using the fourth edition of the Emotional Availability (EA) Scales (Biringen, 2008). EA refers to the capacity of a dyad to share an emotionally healthy relationship and is a concept founded in attachment theory. An EA framework (as operationalized by the EA scales) broadens the concept to include “emotional” and “dyadic” features of the relationship, partly by including codes that capture the infant's contribution to the relational interaction. The measure has been found to be reliable and valid across contexts, and has been consistently predictive of attachment categories, regardless of the context of EA assessment (Biringen, Derscheid, Vliegen, Closson, & Easterbrooks, 2014).

The coding scheme includes scales measuring the behavior and affect of the parent and the infant. The parent was coded on four dimensions. *Sensitivity* focuses on dyadic expressions of emotions and is a measure of emotional sensitivity as well as behavioral sensitivity. The scale assesses the appropriateness of the parent's affect, clarity of perceptions and appropriate responding to the infant's signals, flexibility of attention and behavior, attunement to timing, ability to resolve conflicts, and parental acceptance of the infant. *Structuring* assesses the extent to which the parent appropriately guides, supports learning, and scaffolds the infant's activities in a way that engages the infant in sustained interactions, while permitting a degree of autonomy, meeting the infant at their current level of understanding and using verbal and nonverbal strategies. *Nonintrusiveness* measures the parent's ability to follow the infant's lead during play without interfering, overdirecting, and overstimulating. *Nonhostility* assesses the parent's ability to regulate their own negative emotions and avoid expressing covert hostility (e.g., boredom, frustration) or overt hostility (e.g., aggression, negative statements) to the infant. The infant was coded on two dimensions. *Child responsiveness to the parent* assesses the infant's emotional and behavioral responsiveness to the parent and considers the infant's affect, their responsiveness, age‐appropriate autonomy seeking, physical positioning, lack of overresponsiveness, and avoidance. *Child involvement of the parent* measures the infant's ability to involve the parent in the interaction and considers the infant's attempts to initiate interaction and any evasiveness apparent from body language, gaze, or a lack of engagement.

Each EA dimension includes seven items. Two items are coded on 7‐point scales (from 1 *nonoptimal* to 7 *optimal*), and five items are coded on 3‐point scales (from 1 *nonoptimal* to 3 *optimal*). Scores for all items on each dimension are summed to give a Total Score out of 29 on each dimension, with higher scores indicating more optimal functioning.

To establish interrater reliability, 45 (33%) randomly selected mothers’ videos were coded by a second rater. The intraclass correlation coefficients for *sensitivity, structuring, nonintrusiveness, nonhostility, child responsiveness,* and *child involvement* were .94, .85, .94, .88, .93 and .82, respectively.

### Analysis Plan

Differences between family types on outcome variables were assessed using multivariate analyses of variance (MANOVAs) and independent samples *t* tests. For MANOVAs, correlations between dependent variables were carried out to check for multicollinearity, and the assumption of homogeneity of variance–covariance matrices was tested using Box's test.

#### Covariates

Egg donation and IVF families differed in several demographic (mother's age, father's age, number of children) and fertility treatment variables (number of years since started trying to conceive the child, number of IVF cycles). Typically these variables would be included in the analyses as covariates. However, in the current sample, these variables are systematically related to the defining characteristics of the group. For example, egg donation parents are known to be older parents (Golombok et al., 2005). As such, the potential covariates were viewed as a meaningful, substantial part of the analysis (Miller & Chapman, 2001) and were not included as covariates. Only where a significant difference was found between groups in an outcome variable, and a significant relation existed between a covariate and the outcome variable, was a covariate used. Covariates were used in these cases to gain a greater understanding of whether the difference between groups genuinely reflected the effect of family type or could be explained instead by one of the covariates.

The age of the child differed significantly between family types, and this was an artifact of the recruitment process rather than a defining characteristic of the groups. Correlations were carried out to establish whether any relations existed between child age and the outcome variables. Where significant relations existed, child's age was controlled for in the analyses. Child's age was positively correlated with scores on the following variables: PDI child anger (mothers’ and fathers’ ratings), PDI child controlling (mothers’ and fathers’ ratings), EA Mother Structuring, EA Mother Nonintrusiveness, EA Child Responsiveness (with mother), and EA Child Involvement (with mother).

#### Twin Data

One twin was randomly selected for data analysis. However, in order to check that the inclusion of twin data was not significantly altering the findings, all analyses were rerun without the twin data and are also presented.

## Results

### Quality of Mother–Infant Relationship: Representation of Relationship

#### Representations of Self as a Parent

The PDI Parent Affective Experience variables were entered into a multivariate analysis of variance (MANOVA). Pillai's trace was significant, *F*(1, 146)* *=* *1.93, *p *=* *.03. Univariate tests revealed a significant difference between family types for confidence, *F*(1, 146)* *=* *5.09, *p *=* *.03, *d *=* *−0.38, reflecting lower levels of confidence among egg donation mothers than IVF mothers. Univariate tests for all other variables were nonsignificant (Table 2).

**Table 2:** Means, *SD*,* F*,* p*,* d*, and 95% CI Values for Comparisons of Mothers’ Parent Development Interview Scores Between Family Types

	Egg donation (*N* = 85)		IVF (*N* = 63)		*F*(1, 146)	*p*	*d*	95% CI
	*M*	*SD*	*M*	*SD*				
Representation: self								
Degree of anger	1.66	0.56	1.68	0.58	0.03	.87	−0.04	[−0.36, 0.29]
Expression of anger	1.27	0.47	1.34	0.55	0.84	.36	−0.14	[−0.47, 0.19]
Support: level of need	1.87	0.58	1.98	0.58	1.53	.22	−0.19	[−0.52, 0.14]
Support: satisfaction	3.58	0.59	3.68	0.51	1.31	.25	−0.18	[−0.51, 0.15]
Guilt	1.77	0.64	1.93	0.57	2.59	.11	−0.26	[−0.59, 0.07]
Joy	3.50	0.51	3.55	0.57	0.28	.60	−0.09	[−0.42, 0.23]
Competence	3.41	0.53	3.43	0.53	0.07	.80	−0.04	[−0.36, 0.29]
Confidence	3.23	0.60	3.44	0.49	5.09	.03	−0.38	[−0.71, −0.05]
Level of child focus	3.34	0.65	3.41	0.63	0.42	.52	−0.11	[−0.44, 0.22]
Disappointment	1.08	0.33	1.13	0.46	0.47	.49	−0.13	[−0.45, 0.20]
Warmth	3.55	0.52	3.57	0.57	0.04	.84	−0.04	[−0.36, 0.29]
Attachment awareness	3.35	0.52	3.40	0.60	0.30	.59	−0.09	[−0.42, 0.24]
Hostility	1.04	0.15	1.04	0.19	0.03	.87	0	[−0.33, 0.33]

	*EMM*	*SE*	*EMM*	*SE*	*F*(1, 145)	*p*	*d*	95% CI
Representation: child								
Child aggression	1.40	.05	1.43	.06	0.13	.72	−0.06	[−0.39, 0.26]
Child happiness	3.43	.06	3.60	.07	3.36	.07	−0.31	[−0.63, 0.02]
Child controlling	1.35	.05	1.26	.05	1.40	.24	0.21	[−0.12, 0.53]
Child affectionate	3.43	.07	3.52	.08	0.60	.44	−0.14	[−0.47, 0.19]
Child rejecting	1.07	.03	1.09	.04	0.10	.76	−0.07	[−0.39, 0.26]

	*M*	*SD*	*M*	*SD*	*t*(146)	*p*	*d*	95% CI
Global codes								
Reflective functioning	3.14	0.66	3.28	0.51	−1.49^a^	.14	−0.23	[−0.56, 0.09]
Coherence	3.39	0.56	3.44	0.52	−0.53	.60	−0.09	[−0.42, 0.23]
Richness of perceptions	3.25	0.69	3.31	0.62	−0.57	.57	−0.09	[−0.42, 0.24]

Note

IVF = in vitro fertilization; *EMM* = estimated marginal means.

a *df *= 145.75.

In order to examine whether differences between family types in mothers’ confidence in their parenting ability may have resulted from differences in demographic or fertility treatment variables, covariates were examined. The only variable that approached a significant correlation with confidence was mother's age (*r *=* *−.15, *p *=* *.07). Mother's age was added as a covariate to determine whether the difference in mothers’ representations of their levels of confidence was a genuine effect of family type. With mother's age in the analysis, the test was no longer significant, *F*(1, 145)* *=* *2.23, *p *=* *.14, indicating that egg donation mothers’ representations of themselves as less confident parents was associated with their older age.

#### Representations of the Child

The PDI Child Affective Experience variables were entered into a multivariate analysis of covariance (MANCOVA), with child age included as a covariate. The MANCOVA was nonsignificant, *F*(1, 145)* *=* *1.13, *p *=* *.35. Mothers did not differ in their representations of child anger, happiness, control, affection, or rejection (Table 2).

#### Global Codes

The PDI global codes were analyzed using independent samples *t* tests. The *t* tests were nonsignificant for all of the global codes (Table 2). Egg donation and IVF mothers did not differ in their levels of reflective functioning, the coherence of their representations, or their richness of perceptions.

### Quality of Father–Infant Relationship: Representation of Relationship

#### Representations of Self as a Parent

Fathers’ PDI Parent Affective Experience variables were entered into a MANOVA. Pillai's trace was nonsignificant, *F*(1, 100)* *=* *0.57, *p *=* *.87. Egg donation and IVF fathers did not differ in any of variables assessing their representations of themselves as a parent (Table 3).

**Table 3:** Means, *SD*,* F*,* p*,* d*, and 95% CI Values for Comparisons of Fathers’ Parent Development Interview Scores Between Family Types

	Egg donation (*N* = 67)		IVF (*N* = 35)		*F*(1, 100)	*p*	*d*	95% CI
	*M*	*SD*	*M*	*SD*				
Representation: self								
Degree of anger	1.60	0.60	1.70	0.61	0.58	.45	−0.17	[−0.58, 0.24]
Expression of anger	1.28	0.50	1.33	0.53	0.25	.62	−0.10	[−0.51, 0.31]
Support: level of need	1.52	0.52	1.64	0.58	1.15	.29	−0.22	[−0.63, 0.19]
Support: satisfaction	3.73	0.47	3.67	0.59	0.31	.58	0.12	[−0.29, 0.53]
Guilt	1.64	0.55	1.57	0.52	0.39	.53	0.13	[−0.28, 0.54]
Joy	3.43	0.56	3.36	0.72	0.34	.56	0.11	[−0.30, 0.52]
Competence	3.19	0.63	3.23	0.60	0.11	.75	−0.07	[−0.47, 0.34]
Confidence	3.28	0.50	3.30	0.44	0.03	.87	−0.04	[−0.45, 0.37]
Level of child focus	3.13	0.71	3.20	0.70	0.20	.66	−0.10	[−0.51, 0.31]
Disappointment	1.04	0.18	1.11	0.39	1.89	.17	−0.26	[−0.67, 0.15]
Warmth	3.45	0.55	3.39	0.61	0.27	.60	0.11	[−0.30, 0.51]
Attachment awareness	3.07	0.63	3.11	0.56	0.14	.71	−0.07	[−0.48, 0.34]
Hostility	1.07	0.23	1.04	0.19	0.29	.59	0.14	[−0.27, 0.55]

	*EMM*	*SE*	*EMM*	*SE*	*F*(1, 99)	*p*	*d*	95% CI
Representation: child								
Child aggression	1.31	.06	1.42	.09	0.76	.39	−0.22	[−0.63, 0.19]
Child happiness	3.33	.07	3.42	.10	0.51	.48	−0.16	[−0.57, 0.25]
Child controlling	1.27	.05	1.31	.07	0.16	.69	−0.10	[−0.51, 0.31]
Child affectionate	3.22	.07	3.28	.09	0.29	.59	−0.11	[−0.52, 0.30]
Child rejecting	1.13	.04	1.09	.06	0.22	.64	0.12	[−0.29, 0.53]

	*M*	*SD*	*M*	*SD*	*t*(100)	*p*	*d*	95% CI
Global codes								
Reflective functioning	3.03	0.74	3.01	0.66	0.11	.92	0.03	[−0.38, 0.44]
Coherence	3.28	0.60	3.41	0.49	−1.17	.24	−0.23	[−0.64, 0.18]
Richness of perceptions	3.07	0.76	3.01	0.73	0.34	.74	0.08	[−0.33, 0.49]

Note

IVF = in vitro fertilization; *EMM* = estimated marginal means.

#### Representations of the Child

The PDI Child Affective Experience variables were analyzed using a MANCOVA, with child's age included as a covariate. Pillai's trace was nonsignificant, *F*(1, 99)* *=* *0.34, *p *=* *.89. Fathers in egg donation and IVF families did not differ on any of the representation of the child variables (Table 3).

#### Global Codes

Fathers’ global codes were analyzed using independent samples *t* tests. The *t* tests were nonsignificant for all of the codes. Fathers in egg donation and IVF families did not differ in their levels of reflective functioning, the coherence of their representations, or their richness of perceptions (Table 3).

### Quality of Parent–Infant Relationship: Representations of Relationship (Singleton Only Births)

All analyses for mother–infant and father–infant relationship quality assessed at the representational level were rerun without the data from families with twins in order to check that the inclusion of twin data was not significantly altering the findings. Findings for comparisons between egg donation and IVF families for mothers’ and fathers’ representations of themselves, their child, and their global code scores did not change when the data from twin families were omitted.

### Quality of Parent–Infant Relationship: Observational Assessment

#### Mother–Infant Interaction Quality

As preliminary analyses had found significant correlations between child age and four of the six EA dimensions, child age was controlled for in the following analyses. The EA dimensions of sensitivity, structuring, nonintrusiveness, nonhostility, child responsiveness, and child involvement were entered into a MANCOVA with child's age included as a covariate. Pillai's trace was significant, *F*(1, 132)* *=* *2.40, *p *=* *.03. One‐way analyses of covariance (ANCOVAs) found a significant difference between family types for mothers’ sensitivity, *F*(1, 132)* *=* *5.62, *p *=* *.02, *d *=* *−0.44, and mothers’ structuring, *F*(1, 132)* *=* *7.79, *p *=* *.01, *d *=* *−0.51, reflecting less optimal sensitivity and structuring among egg donation mothers. The ANCOVAs for child responsiveness and child involvement were also significant, child responsiveness, *F*(1, 132)* *=* *7.68, *p *=* *.01, *d *=* *−0.50, child involvement, *F*(1, 132)* *=* *7.43, *p *=* *.01, *d *=* *−0.50, indicating less optimal responsiveness and involvement among egg donation infants. Univariate test values and estimated marginal means for groups are presented in Table 4.

**Table 4:** Estimated Marginal Means (*EMM*), *SE*,* F*,* p*,* d*, and 95% CI Values for Comparisons of Mothers’ and Infants’ Scores on the EA Dimensions Between Family Types

	Egg donation (*N* = 76)		IVF (*N* = 59)		*F*(1, 132)	*p*	*d*	95% CI
	*EMM*	*SE*	*EMM*	*SE*				
Mother								
Sensitivity	23.26	.47	25.04	.54	5.62	.02	−0.44	[−0.78, −0.09]
Structuring	22.73	.41	24.54	.47	7.79	.01	−0.51	[−0.85, −0.16]
Nonintrusiveness	22.56	.53	23.29	.61	0.74	.39	−0.16	[−0.50, 0.18]
Nonhostility	26.97	.24	27.31	.27	0.76	.39	−0.16	[−0.51, 0.18]
Infant								
Responsiveness	22.80	.52	25.09	.60	7.68	.01	−0.50	[−0.85, −0.16]
Involvement	21.14	.48	23.21	.55	7.43	.01	−0.50	[−0.84, −0.15]

Note

EA = emotional availability; IVF = in vitro fertilization.

#### Father–Infant Interaction Quality

Fathers’ and infants’ scores on the six EA dimensions were entered into a MANOVA. Pillai's trace was nonsignificant, *F*(1, 84)* *=* *0.41, *p *=* *.87. Egg donation fathers and infants did not differ from IVF fathers and infants on any of the EA dimensions (Table 5).

**Table 5:** Means, *SD*,* F*,* p*,* d*, and 95% CI Values for Comparisons of Fathers’ and Infants’ Scores on the EA Dimensions Between Family Types

	Egg donation (*N* = 55)		IVF (*N *= 31)		*F*(1, 84)	*p*	*d*	95% CI
	*M*	*SD*	*M*	*SD*				
Father								
Sensitivity	23.93	3.31	23.84	3.80	0.01	.91	0.03	[−0.04, 0.47]
Structuring	23.40	3.45	23.42	3.35	0.001	.98	−0.01	[−0.45, 0.43]
Nonintrusiveness	22.33	3.95	22.23	5.06	0.01	.92	0.02	[−0.42, 0.46]
Nonhostility	27.62	1.53	27.19	1.92	1.26	.26	0.26	[−0.19, 0.70]
Infant								
Responsiveness	23.72	3.76	23.90	4.41	0.04	.85	−0.05	[−0.49, 0.40]
Involvement	22.80	3.94	22.97	4.72	0.03	.86	−0.04	[−0.48, 0.40]

Note

EA = emotional availability; IVF = in vitro fertilization.

### Quality of Parent–Infant Interaction (Singleton Only Births)

When mother–infant interaction analyses were rerun without twin data, Pillai's trace was nonsignificant, *F*(1, 114)* *=* *0.89, *p *=* *.50. The univariate ANCOVAs indicated that none of the group comparisons was significant. Univariate test statistics and estimated marginal means are presented in Table 6. There was no difference between egg donation mother–infant dyads and IVF mother–infant dyads for any of the EA dimensions when only data from singleton infants were considered. The analyses without data from twin families suggest that the poorer quality of mother–infant interaction found in egg donation families in the full sample can be somewhat explained by the inclusion of families with twins.

**Table 6:** Estimated Marginal Means (*EMM*), *SE*,* F*,* p*,* d*, and 95% CI Values for Comparisons of Singleton Mothers’ and Infants’ Scores on the EA Dimensions Between Family Types

	Egg donation (*N* = 66)		IVF (*N* = 51)		*F*(1, 114)	*p*	*d*	95% CI
	*EMM*	*SE*	*EMM*	*SE*				
Mother								
Sensitivity	23.50	.49	24.66	.57	2.16	.14	−0.29	[−0.66, 0.08]
Structuring	23.10	.42	24.24	.48	2.89	.09	−0.34	[−0.70, 0.03]
Nonintrusiveness	22.39	.58	23.10	.67	0.58	.45	−0.15	[−0.52, 0.22]
Nonhostility	26.94	.26	27.30	.29	0.78	.38	−0.17	[−0.54, 0.19]
Infant								
Responsiveness	23.21	.54	24.75	.63	3.07	.08	−0.35	[−0.72, 0.02]
Involvement	21.43	.52	23.05	.60	3.74	.06	−0.38	[−0.75, −0.01]

Note

EA = emotional availability; IVF = in vitro fertilization.

When father–infant interaction analyses were rerun without data from twin families, none of the findings changed.

## Discussion

The purpose of this study was to examine the quality of parent–infant relationships in families formed using egg donation, in which mothers and infants do not share a genetic connection, and a comparison group of families who conceived following IVF treatment with their own gametes. When the quality of the parent–infant relationship was assessed at the representational level, very few differences were found between egg donation and IVF parents. Differences were found between egg donation and IVF families in the observational assessment of parent–infant relationship quality, indicating less optimal interaction quality in egg donation families. These differences were found only between mothers and infants, and only when data from twin families were included.

Parental representations of the parent–child relationship are important predictors of future outcomes, including mother–infant attachment security (Zeanah et al., 1994) and child attachment security (George & Solomon, 1996). In the current study, mothers in both family types viewed themselves as high in warmth and joy, moderate to high in child focus and competence, and low in disappointment and anger. Egg donation and IVF mothers both represented their children as affectionate and happy, with low levels of angry, rejecting, or controlling behaviors. Mothers also showed moderate levels of reflective functioning, coherence, and richness of perceptions. Similarly, egg donation and IVF fathers had representations of themselves that were moderate to high in warmth, joy, and confidence, and low in disappointment and anger, with representations of the child as affectionate and happy and neither rejecting nor controlling. Viewed within an attachment framework, parents’ representations of the parent–infant relationship indicate a high quality of relationship in both family types and suggest probable positive future attachment‐related outcomes.

That egg donation and IVF parents’ representations of the parent–child relationship were found to be more similar than different is in line with findings from the second phase of the UK Longitudinal Study of Assisted Reproduction Families when children were aged 2, in which genetically unrelated dyads had largely similar representations of the parent–child relationship to genetically related dyads when assessed using the PDI (Golombok et al., 2005). The findings diverge, however, in that egg donation mothers at age 2 showed higher levels of joy than genetically related mothers, a difference that was not present in the current sample. The only difference found in the current sample between family types in parents’ representations of the parent–infant relationship was between egg donation and IVF mothers, with egg donation mothers showing lower confidence in their parenting ability than IVF mothers, a factor that seemed to be associated with egg donation mothers’ older age, rather than being an effect of family type. This is in contrast to qualitative studies that have found that older mothers conceptualize their age as beneficial to their parenting (Mac Dougall, Beyene, & Nachtigall, 2012) and is more in line with Hershberger's (2007) finding that pregnant egg recipients stated concerns about the effects of advanced maternal age on motherhood. It should be noted, however, that mothers in both family types showed mean confidence scores in the moderate‐high range, so neither groups’ scores were a cause for concern.

The positive representations of the parent–child relationship found in both family types is not surprising when considered in light of research highlighting the risk factors that can negatively affect maternal representations. Known risk factors include depressive symptoms (Rosenblum et al., 2002), lack of support from partners, and lack of social support (Vreeswijk, Rijk, Maas, & van Bakel, 2015). Parents in the present study could be considered a low‐risk sample; they were highly satisfied with the support available to them (as assessed by the PDI), and it has also been suggested that couples who persist with fertility treatment despite failures may comprise a group of self‐selected individuals with strong coping skills who may be less affected by the everyday hassles of parenthood (McMahon, Gibson, Leslie, Cohen, & Tennant, 2003). Having waited so long to have their child, parents who have formed their families through assisted reproduction may be particularly committed and loving parents, who view their child as especially precious (Golombok et al., 2005), and this is likely reflected in their positive representations of the parent–infant relationship.

With regard to parent–infant interaction quality, differences emerged between family types on several of the EA dimensions for mother–infant interactions only. Specifically, egg donation mothers were less optimally sensitive and structuring than IVF mothers, and egg donation infants were less emotionally responsive and involving of the mother than IVF infants. These differences were of a medium effect size. To put the sample's scores into context, however, egg donation and IVF mothers’ and infants’ mean scores on all EA dimensions were at the upper end of the scales, indicating good relationship quality. Specifically, mothers’ interactions in both groups were sensitive and appropriately structuring, and infants were emotionally responsive and appropriately involving of their mothers. Mothers in both groups showed particularly low hostility toward their infants (i.e., no signs of boredom or discontent), which has been found to be a feature of parent–infant interactions where parents have wanted to have children for a long time and are happy to be parents (McMahon et al., 2003).

The finding that egg donation mothers were less sensitive in their interactions with their infants merits further investigation, due to both the causal role of maternal sensitivity in the development of infant attachment security (Verhage et al., 2016) and its relations to other socioemotional and cognitive outcomes (Leerkes, Blankson, & O'Brien, 2009). That a less optimal quality of mother–infant interaction was found among egg donation families could be viewed as contradicting previous findings that indicated more positive parent–child relationships among egg donation families in infancy and early childhood compared with natural conception families (Golombok et al., 2004, 2005, 2006). The present study, however, differed from previous studies in several important ways. First, the present study is the only one to control for the use of IVF, allowing for comparisons between genetic and nongenetic parenthood without the confounding factors of experience of infertility and fertility treatment. Second, the current study is the only one to examine parent–child relationship quality in the early years using an observational measure. The interview measures used in prior studies may not have been sensitive enough to detect differences between groups. Observational measures provide more detailed examinations of the dynamic nature of parent–infant interactions, in which it may be more difficult for participants to present themselves in a socially desirable manner (Kerig, 2001). Third, the current study examines families formed through identity‐release donation rather than anonymous donation, and donation type may have affected mother–infant relationship quality. Although it is not possible to tease apart whether egg donation mother–infant dyads’ poorer results were related to egg donation more generally, or identity‐release donation specifically, as to do so would require a direct comparison between families who had used identity‐release donation and anonymous donation, both explanations remain possible.

It was hypothesized that infancy is the stage at which differences in parent–infant relationship quality between egg donation and IVF parents are most likely to occur. Although the transition to parenthood poses challenges for all parents (Cowan & Cowan, 1992), the transition to adoptive parenthood literature suggests that nongenetic parents may face additional challenges, including concerns about parenting a genetically unrelated child, awareness of societal stigma regarding nongenetic parenthood, and uncertainties about parental role definition (Goldberg et al., 2011; Kirk, 1964). Egg donation parents may face similar challenges, and it is possible that the differences seen between egg donation and IVF mothers in the current sample reflect these challenges. The process of feeling comfortable with the parental role may take longer for egg donation mothers than for IVF mothers, who do not have to manage the idea of nongenetic parenthood. Concerns about parental role definition may be compounded for some mothers by the use of identity‐release donation as they may perceive the donor as a threat (Lampic et al., 2014). Qualitative data from the present study exploring egg donation mothers’ perceptions of the significance of egg donation in the developing mother–infant relationship, and their feelings about the identity‐release process, suggest that the process of “claiming” the child as their own was a complex and highly individual process, which some mothers found to be challenging (in preparation). Research with egg donation mothers of older children suggests that mothers may feel more comfortable with nongenetic motherhood over time (Kirkman, 2008). It is possible that the subtle differences seen in the present sample's interactions capture a moment in the mother–infant relationship where egg donation mothers and infants are still “finding their feet” and adjusting to the new relationship. Whether these differences remain over time will only be addressed through longitudinal research with the current sample.

It is worth noting, however, that when observational measures were used in the UK Longitudinal Study of Assisted Reproduction Families to examine parent–child relationship quality at age 7, less positive mother–child interactions were found among egg donation dyads in comparison with sperm donation dyads (where mothers and children share a genetic connection; Blake, 2011). These findings have been explained to some extent by whether parents had disclosed the donor conception to the child, with significant interactions found between family type and disclosure status (Blake, 2011; Golombok et al., 2011). Specifically, disclosing egg donation dyads scored lower on mother–child mutuality than disclosing sperm donation dyads, but there were only small differences in mutuality between family types in nondisclosing dyads. As disclosure intentions in infancy often do not match later disclosure decisions (Applegarth, Kaufman, Josephs‐Sohan, Christos, & Rosenwaks, 2016), analyses of mother–infant relationship quality based on disclosure intentions were not considered useful at this developmental stage but may merit consideration at later stages. Similarly, when children in the UK Longitudinal Study of Assisted Reproduction Families were aged 14, egg donation mothers and adolescents were found to have poorer relationship quality than sperm donation mother–adolescent dyads when assessed using questionnaire measures of family relationship problems and parental acceptance/rejection (Golombok et al., 2017). Although no differences were found at age 14 in the observational assessment of mother–adolescent interaction quality, taken together with the present study, these findings may hint at a pattern of subtle differences indicating less optimal relationship quality between egg donation mothers and children in comparison to genetically related dyads.

Interestingly, when data from twin families were omitted from the current sample, no statistically significant differences were found between egg donation and IVF mother–infant dyads, although the data showed a similar trend toward less optimal relationship quality in egg donation dyads for between‐group comparisons of maternal structuring, infant responsiveness, and infant involvement. Although this may be a consequence of reduced statistical power, it is also possible that being a mother of twins may affect family functioning differently in different family types. Parenting twins is known to negatively affect parental adjustment during the transition to parenthood (Vilska et al., 2009). It is possible that being the parent of twins may have proved more challenging for egg donation mothers, and this was reflected in their interaction scores. Although the number of twin mothers in the current sample is too small to draw firmer conclusions, whether twin parenthood is a risk factor for less optimal interactions among egg donation mothers and infants merits further investigation with larger samples as twin births are a common outcome of fertility treatment (Centers for Disease Control and Prevention, 2017).

A limitation of the study is that no information is available about the families who declined to return reply slips to the researchers, so it is not possible to ascertain whether these families differed from those who contacted the researchers. As families had been guaranteed that clinics had no knowledge of who participated in the study, it was not possible to obtain information about nonresponders without compromising the anonymity of participants. Previous studies of gamete donation families have found that parents who declined study participation often cited concerns around participation jeopardizing nondisclosure decisions or not wanting to be reminded about the lack of genetic relationships within families (Golombok, Cook, Bish, & Murray, 1995). It has also been suggested that nondisclosing families may be the least likely to participate in gamete donation studies (Nachtigall, Tschann, Szkupinski Quiroga, Pitcher, & Becker, 1997). Even so, the current sample included some families who had decided not to disclose their treatment type to the child or others.

A further limitation is that not all fathers participated in the study, with fewer IVF than egg donation fathers taking part. It is possible that as egg donation children were more likely to be the first child in the household and parents had been trying longer to conceive them, egg donation fathers may have been more motivated to take part in research than IVF fathers. Statistical power was, thus, lower for comparisons between egg donation and IVF fathers. Although the sample size was sufficient to detect a large effect at α = .05 (Cohen, 1992), it is possible that small differences between family types may not have been detected. For the analyses comparing parent–child relationship quality between egg donation and IVF mothers, sample sizes were adequate to detect a medium effect (Cohen, 1992). Fathers continue to be underrepresented in family‐based research (Doyle, Weller, Daniel, Mayfield, & Goldston, 2016), and future studies would benefit from recruiting larger samples of fathers in order to ensure both that there is adequate power to detect medium to small effect sizes between groups and to provide a more thorough understanding of the family system. Finally, given that the sample was relatively homogenous in terms of parents’ ethnicity, education, and family size, and all lived in the United Kingdom, the findings are limited in their generalizability to other sociocultural contexts.

One of the strengths of the study is the size of the egg donation sample, which is the largest to date in a study utilizing in‐depth interview and observational measures, and can be considered a relatively large sample, particularly given the difficulties involved in recruiting assisted reproduction families to take part in studies involving discussion of sensitive topics, around which there may be real or perceived stigma (Nachtigall et al., 1997). That families were recruited from fertility clinics is also a study strength, as investigations of assisted reproduction families often rely on samples recruited through online registers or support groups, which may not be representative of assisted reproduction families in general.

The current study was the first to examine parent–child relationship quality in egg donation families in infancy at both the representational and observational levels. The assessment of both components provides a more thorough evaluation of the parent–infant relationship than has previously been possible, and from an attachment perspective increases understanding about the organization of the relationship (Korja et al., 2010). The study also offers the first investigation of parent–child relationships in families created through identity‐release egg donation. As an increasingly popular treatment option in the United States and United Kingdom, yet one not without specific concerns, an examination of parent–child relationship quality following this treatment type was long overdue. Furthermore, as recent years have seen a sharp increase in the number of IVF cycles using donor eggs (Centers for Disease Control and Prevention, 2017; Human Fertilisation and Embryology Authority, 2018), the study is particularly timely. That egg donation families showed more similarities than differences to IVF families in infancy should prove reassuring to clinicians, prospective egg donation parents, and families already created in this way. That subtle, yet significant, differences were found between egg donation and IVF mothers and their infants in interaction quality merits further investigation and suggests that nongenetic motherhood may pose particular challenges to some women during early parenthood.
